# iProt-Sub: a comprehensive package for accurately mapping and predicting protease-specific substrates and cleavage sites

**DOI:** 10.1093/bib/bby028

**Published:** 2018-04-19

**Authors:** Jiangning Song, Yanan Wang, Fuyi Li, Tatsuya Akutsu, Neil D Rawlings, Geoffrey I Webb, Kuo-Chen Chou

**Affiliations:** 1 Monash Centre for Data Science, Faculty of Information Technology, Monash University, Melbourne, VIC 3800, Australia; 1a Biomedicine Discovery Institute and Department of Biochemistry and Molecular Biology, Monash University, Melbourne, VIC 3800, Australia and ARC Centre of Excellence in Advanced Molecular Imaging, Monash University, Melbourne, VIC 3800, Australia; 2 Institute of Image Processing and Pattern Recognition, Shanghai Jiao Tong University, and Key Laboratory of System Control and Information Processing, Ministry of Education of China, Shanghai, 200240, China; 3 Biomedicine Discovery Institute and Department of Biochemistry and Molecular Biology, Monash University, Melbourne, VIC 3800, Australia; 4 Bioinformatics Center, Institute for Chemical Research, Kyoto University, Uji, Kyoto, 611-0011, Japan; 5 EMBL European Bioinformatics Institute, Wellcome Trust Genome Campus, Hinxton, Cambridgeshire CB10 1SD, UK; 6 Gordon Life Science Institute, Boston, MA 02478, USA and Center for Informational Biology, School of Life Science and Technology, University of Electronic Science and Technology of China, Chengdu 610054, China

**Keywords:** protease, substrate, cleavage site, sequence analysis, machine learning, five-step rule

## Abstract

Regulation of proteolysis plays a critical role in a myriad of important cellular processes. The key to better understanding the mechanisms that control this process is to identify the specific substrates that each protease targets. To address this, we have developed iProt-Sub, a powerful bioinformatics tool for the accurate prediction of protease-specific substrates and their cleavage sites. Importantly, iProt-Sub represents a significantly advanced version of its successful predecessor, PROSPER. It provides optimized cleavage site prediction models with better prediction performance and coverage for more species-specific proteases (4 major protease families and 38 different proteases). iProt-Sub integrates heterogeneous sequence and structural features and uses a two-step feature selection procedure to further remove redundant and irrelevant features in an effort to improve the cleavage site prediction accuracy. Features used by iProt-Sub are encoded by 11 different sequence encoding schemes, including local amino acid sequence profile, secondary structure, solvent accessibility and native disorder, which will allow a more accurate representation of the protease specificity of approximately 38 proteases and training of the prediction models. Benchmarking experiments using cross-validation and independent tests showed that iProt-Sub is able to achieve a better performance than several existing generic tools. We anticipate that iProt-Sub will be a powerful tool for proteome-wide prediction of protease-specific substrates and their cleavage sites, and will facilitate hypothesis-driven functional interrogation of protease-specific substrate cleavage and proteolytic events.

## Introduction

Proteolytic cleavage is one of the few irreversible posttranslational modifications. It plays a key role in numerous developmental and physiological processes, including digestion, protein degradation, endocrine signaling and cell division [1]. This process is controlled by proteases (also known as peptidases or proteinases) that selectively cleave the peptide bonds between amino acids in specific protein or peptide substrates. Proteases have central roles in ‘life or death’ processes. Through the highly selective proteolytic processing, proteases can precisely regulate a myriad of biological processes across all living organisms [1]. In addition, these are also many other proteases involved in protein degradation rather than processing, for example cathepsin D and cathepsin B. Pepsin, trypsin and chymotrypsin also come into this category, even though they have a defined specificity because they degrade so many substrates, most of which are foreign to the body [2]. The malfunction or deregulation of proteases results in many pathological conditions [3]. For example, proteases are often associated with cancer invasion and metastasis because of their ability to degrade the extracellular matrix [4–8]. Intriguingly, proteases can function as part of an extensive network of proteolytic interactions through interacting with other important signaling pathways involving other protein substrates and enzymes, termed the ‘protease web’ [9].

Our knowledge of the mechanisms that regulate and control the proteolytic processing of proteases remains limited. The precise understanding of the biological function of a protease requires the identification of the complete repertoire of its natural substrates and corresponding substrate cleavage sites [10, 11]. The specificity of proteases can vary significantly, depending on the protease and the active sites, with the cleavage site selectivity ranging from preferences for limited and specific amino acids at specific positions, to more general preferences with little discrimination. Current experimental methods for proteolytic cleavage characterization include one-dimensional and two-dimensional gel-based methods (used for identifying the substrates) [12], N-terminal peptide identification methods (for identifying both substrates and cleavage sites), methods using mass spectrometry, as well as quantitation methods of proteolysis to better understand the dynamics and extent of proteolytic events such as the TAILS method [13]. Despite the advances of these experimental methods, they are labor intensive, expensive and time-consuming, and are often limited to the investigation of one protease each time. In this context, it is highly desirable to develop cost-effective computational methods that can be used to identify the target substrates for a specific protease and to facilitate the characterization of substrate specificity and the function of proteases.

The importance and value of the *in silico* identification of protease target substrates and cleavage sites has led to the development of a variety of computational methods for predicting protease-specific substrates and cleavage sites. A number of computational studies have suggested that substrate cleavage sites (sites surrounding the cleavage P1 sites) targeted by proteases present unique structural and physicochemical properties that vary across different proteases, which can be exploited to predict potential cleavage sites [11, 14–24]. However, the most successful computational methods use a combination of these features with other complementary features [10, 14–16, 25–31], achieving overall accuracies of 70–90% for most of the proteases under investigation. A consensus resulting from such computational methods is that machine learning algorithms that take into consideration integrated heterogeneous information can be used to build more accurate predictive models for most of the proteases under investigation. Often a consensus scoring mechanism is performed using scoring function-based techniques or machine learning techniques. The former includes PeptideCutter [32], PoPS [33] and SitePrediction [34]. The latter has gained significant interest in recent years and includes CASVM [35], Cascleave [15], Pripper [36], Cascleave 2.0 [30], PROSPER [29, 31] and PROSPERous [37]. For the substrate cleavage site prediction of specific proteases, some *ad hoc* consensus schemes can also be effective, including GraBCas [38], CaSPredictor [39] and GPS-CCD [40].

At the end of 2012, we published PROSPER (PROtease substrate SPecificity servER), a bioinformatic tool for predicting target substrates and their specific cleavage sites for 23 proteases [29]. It represented the first comprehensive server capable of predicting cleavage sites of multiple proteases within a single substrate sequence using machine learning techniques. To date, the PROSPER server has attracted >25 000 unique users worldwide and has processed >60 000 job submissions since its inception. Here, we build on this previous work to develop a new computational method, termed iProt-Sub to address the problem of identifying the most probable protease-specific substrates and their detailed cleavage sites from the substrate sequence information. According to the well-known Chou’s five-step rule [41] in developing a useful predictor, we need to accomplish the following: (1) benchmark data set construction, (2) protein sample formulation, (3) operating algorithm, (4) evaluating expected accuracy and (5) Web server establishment. In this work, we have considerably improved the design of iProt-Sub package for each of the five procedures.

More specifically, using a well-prepared benchmark data set, iProt-Sub extracts a wide range of sequence-derived structural, physicochemical and evolutionary information, which is further integrated into a common machine learning framework in the form of support vector machine (SVM) classifiers, to identify and rank potential substrate cleavage sites in a protease-specific manner. The cleavage site prediction models are trained and optimized to achieve best-performing prediction by performing a 5-fold cross-validation test. Benchmarking experiments indicated that the iProt-Sub method compared favorably with recently published methods. Moreover, mapping of the protease-specific cleavage target substrates at the proteome-wide scale was highly accurate and selective. iProt-Sub is accessible through a user-friendly Web application available at http://iProt-Sub.erc.monash.edu/. The Web application of iProt-Sub features a powerful and convenient graphic interface that allows the visualization and analysis of the predicted cleavage site within the same protein by different proteases simultaneously. The implemented iProt-Sub server thus represents a centralized Web resource for accurate *in silico* prediction of protease-specific substrates and their cleavage sites.

## Materials and methods

### Overall workflow of iProt-Sub

**Figure 1:**
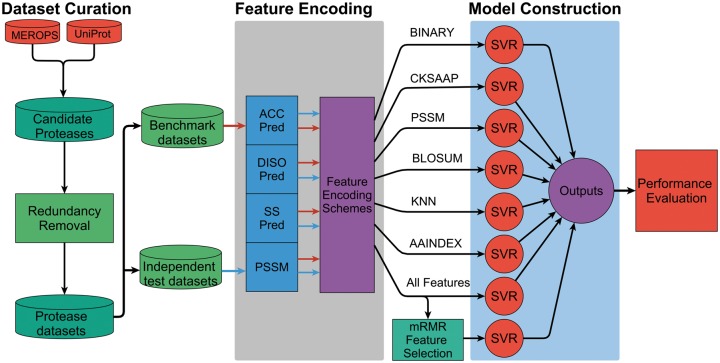
The workflow of the iProt-Sub methodology. There exist four major stages during the development of iProt-Sub, including Data set curation, Feature extraction and encoding, Model construction and Performance evaluation. Refer to the main text for a detailed description of each of the major stages. ‘All features’ included all the 11 types of extracted features (a detailed list is shown in Table 2).

iProt-Sub represents an advanced version of PROSPER [29]. Importantly, the improvement of iProt-Sub over PROSPER is reflected by the following: (1) larger coverage of more proteases. iProt-Sub can be used to predict protease-specific substrates and cleavage sites for 38 different proteases, whereas PROSPER covered 23 proteases; (2) use of a wider range of sequence-derived features. iProt-Sub uses 11 diverse types of sequence-based features (4562-dimensional); (3) application of a more effective feature selection technique to filter out irrelevant and noisy features. iProt-Sub uses the mRMR (minimum redundancy maximum relevance) [42] algorithm to identify more informative features to enhance the predictive performance: (4) improved predictive performance. Through an effective feature extraction, selection and model learning strategy, iProt-Sub consistently achieves improved predictive performance for predicting the substrate cleavage sites for all tested proteases and (5) completely redesigned interface. The new iProt-Sub Web server now provides a more user-friendly and interactive interface that enhances user experience. The overall flowchart of the iProt-Sub methodology is shown in Figure 1.

### Data sets

Numerous studies have suggested that a high-quality, well-established data set is crucial for training a robust and reliable prediction model of protease cleavage sites [37, 43–45]. In this study, we constructed a well-prepared benchmark data set for assessing the predictive performance of our method and other existing methods. For this purpose, we used the MEROPS database [46], which is a comprehensive information resource for proteases, their substrates and inhibitors. Only experimentally verified substrate sequences and cleavage sites were retrieved. The annotations of experimentally verified cleavage sites and the corresponding proteases that cleave the target substrates were extracted from MEROPS, while the annotations of protein identifiers of the substrates and their sequence information were extracted from UniProt [47]. In particular, exopeptidases (aminopeptidases, carboxypeptidases, etc.) and oligopeptidases were generally not included, which is consistent with our previous study [29]. As we are more interested in predicting cleavages within native proteins, peptidases that work at pH extremes and are likely to degrade only denatured proteins were also excluded [29].

To avoid potential model bias and overfitting, we performed sequence clustering and homology reduction using the CD-HIT program [48]. We removed sequence redundancy in the retrieved data set, so that any two sequences in the benchmark data set and independent test data set have a sequence identity of <70%, which is in accordance with previous studies [15, 30, 37, 49, 50]. After this procedure, we only retained those proteases that had ≥50 experimentally verified cleavage sites. Finally, we ended up with 38 proteases with a total of 3688 substrates and 6637 cleavage sites. A complete list of these substrate sequences and their cleavage sites can be found at the iProt-Sub website. A statistical summary of the curated data sets in this study is shown in Table 1.

**Table 1.** Statistical summary of the substrate data sets curated in this study

No	MEROPS ID	Protease name	Number of substrates	Number of cleavage sites	Number	MEROPS ID	Protease name	Number of substrates	Number of cleavage sites
1	A01.009	Cathepsin D	23	59	20	M16.002	Insulysin	6	50
2	C01.032	Cathepsin L	17	63	21	S01.010	Granzyme B (human-type)	410	515
3	C02.001	Calpain-1	30	61	22	S01.017	Kallikrein-related protease 5	31	59
4	C02.002	Calpain-2	17	66	23	S01.131	Elastase-2	45	133
5	C14.003	Caspase-3	251	373	24	S01.135	Granzyme A	44	57
6	C14.004	Caspase-7	48	64	25	S01.136	Granzyme B (rodent-type)	143	157
7	C14.005	Caspase-6	58	165	26	S01.139	Granzyme M	491	707
8	C14.009	Caspase-8	37	56	27	S01.233	Plasmin	42	89
9	M10.001	Matrix metallopeptidase-1	21	52	28	S01.251	Kallikrein-related peptidase 4	78	80
10	M10.002	Matrix metallopeptidase-8	23	85	29	S08.071	Furin	56	75
11	M10.003	Matrix metallopeptidase-2	35	115	30	A01.009 (mouse)	Cathepsin D	342	579
12	M10.004	Matrix metallopeptidase-9	43	290	31	A01.010 (mouse)	Cathepsin E	655	1216
13	M10.005	Matrix metallopeptidase-3	44	132	32	C14.001 (mouse)	Caspase-1	47	53
14	M10.008	Matrix metallopeptidase-7	42	142	33	S01.010	Granzyme B (human-type)	77	88
15	M10.009	Matrix metallopeptidase-12	23	178	34	S01.136	Granzyme B (rodent-type)	168	201
16	M10.013	Matrix metallopeptidase-13	23	90	35	S08.073	PCSK2 peptidase (mouse)	21	68
17	M10.014	Membrane-type matrix metallopeptidase-1	36	92	36	S08.109	KPC2-type peptidase (*Caenorhabditis elegans*)	34	115
18	M12.221	ADAMTS4 peptidase	13	50	37	S26.001	Signal peptidase I (*Escherichiacoli*)	141	141
19	M13.001	Neprilysin	19	67	38	S26.001	Signal peptidase I (*Salmonella typhimurium*)	54	54

In this study, five of the six of the substrate sequences in the resulting data set obtained above were randomly selected as the benchmark training data set, while the remaining one of the six of the data set was used as the independent test data set. The purpose of constructing a benchmark training data set was to optimize the parameters of machine learning algorithms, train the prediction model and evaluate model performance in an *n*-fold cross-validation manner, whereas the purpose of constructing the independent test data set was to validate the generalization ability of trained prediction models and compare them with other existing tools. None of the substrate sequences in this constructed independent test data set appeared in the benchmark data set, which ensures that a fair assessment of model performance can be achieved.

### Positive and negative samples

In this study, the number of negative samples (i.e. non-cleavage sites) in the data set of protease-specific substrate cleavage sites greatly dominates the number of the positive samples (i.e. cleavage sites). This leads to a class imbalance problem. If not addressed, this can result in models that favor negative predictions over positive [29, 51]. To address this data imbalance issue, we used a down-sampling strategy, randomly discarding from the overrepresented negative samples, to impose a ratio of 1 positive to every 3 negatives, as previously suggested [15, 25, 29, 51, 52].

To extract the sequence-based features of positive and negative samples, we used a local sliding window approach, with a fixed window size of P8–P8′ sites (i.e. eight residues in the upstream and another eight residues in the downstream to surround the cleavage site). The overall size of the sliding window was 16 sites. With regard to the selection of reliable negative samples, several previous studies have indicated that a few cleavage sites at the P1 position were predicted to be solvent inaccessible [14–16]. In view of these studies and for the purpose of extracting reliable non-cleavage sites, we randomly selected those negative samples with P1 sites predicted as solvent inaccessible by the SABLE program [53] for constructing the prediction models.

### Sequence encoding schemes

We formulate cleavage site prediction as a classification problem and solve it using machine learning techniques. Each potential cleavage site (or non-cleavage site) of an amino acid sequence is represented by a feature vector *x* with *D*-dimensional feature components {*x*_1_, …, *x_D_*}. The problem is to predict the label *y* of the site of interest that is represented and encoded by *D*-dimensional features. The *y* will be defined as ‘1’ if the site is a cleavage site for a protease, and ‘0’ otherwise.

The representation form of a potential site is determined based on the so-called ‘sequence encoding scheme’, which is used for extracting the potentially useful information from the amino acid sequence (often combined with predicted structural information) and converting the sequence data into numerical feature vectors [54, 55]. Accordingly, the sequence-encoding scheme plays a crucial role in determining the predictive performance of the machine learning-based model. In this study, we derived a great variety of features organized into 11 different types. We evaluated the relative predictive performance to identify effective combinations of features that could lead to the overall best predictive performance for a given protease. In addition to sequence-derived features, we also integrated evolutionary, physicochemical properties and predicted structural features. Detailed information on the software or databases we used to extract these different types of features is listed in Table 2, along with the feature category, annotations, dimension and references. Below we will first describe the different types of sequence encoding schemes in detail, and then describe the SVM learning algorithm that is used to train the prediction models to predict *y* given *x*.

**Table 2.** A complete list of sequence-derived structural, physicochemical and evolutionary features used

Number	Category	Feature type	Annotation	Dimension	Tool/database	Reference
1	Sequence-derived	BINARY	Binary sequence profile features	336	–	[37, 51]
2		CKSAAP	Composition of *k*-spaced amino acid pair	2400	–	[54, 56–59]
3		KNN	*k*-nearest neighbor features of local sequences	5	–	[58, 60]
4		AAC	Composition of 20 amino acid types	20	–	–
5	Evolutionary	PSSM	Position-specific scoring matrix	320	PSI-BLAST	[61]
6		BLOSUM	BLOSUM62 matrix	336	–	[62]
7	Physicochemical property	AAIndex	Numerical indices representing various physicochemical and biochemical properties of amino acids and pairs	1024	AAIndex	[63]
8		CHR	Charge/hydrophobicity ratio	9	AAIndex	[63]
9	Structural	SS	Predicted secondary structure	48	SABLE	[53]
10		SA	Predicted solvent accessibility	32	SABLE	[53]
11		DISO	Predicted natively disordered region	32	DISOPRED2	[64]
Total				4562		

*Note:* A local window size of 16 amino acid residues was used to extract the features. The last row shows the total number of features used.

#### Sequence or sequence-derived features

With the avalanche of protein sequences generated in the post-genomic era, one of the most challenging problems in computational biology is how to express a biological sequence with a discrete model or a vector, yet still keep considerable sequence-order information or key pattern characteristic. This is because all the existing machine learning algorithms can only handle vectors but not sequences, as elucidated in [65]. However, a vector defined in a discrete model may completely lose all the sequence-pattern information. To avoid this for proteins, the pseudo amino acid composition (AAC) [66] or PseAAC [67] was proposed. Ever since the concept of PseAAC was proposed, it has been widely used in nearly all the areas of computational proteomics (see, e.g., [68–70] as well as a long list of references cited in [71]). According to the concept of general PseAAC [41], a protein sequence can be formulated as:

(1) $$P={\left[\Psi_{1}\;\Psi_{2}\;\cdots \;\Psi_{u}\;\cdots \;\Psi_{\Omega }\right]}^{T},$$

where *T* is a transpose operator, while the subscript Ω is an integer and its value as well as the components $\Psi_{u}\;(u=1,\;2,\;\cdots ,\;\Omega )$ depend on the way to extract the desired information from the amino acid sequence of *P*, as done in a series of recent publications (see, e.g., [72–78]).

Encouraged by the success of using PseAAC to deal with protein/peptide sequences, the concept of PseKNC (pseudo K-tuple nucleotide composition) [79, 80] was developed for generating various feature vectors for DNA/RNA sequences [81, 82] that have proved useful as well [83–90]. Particularly, recently a powerful Web server called ‘Pse-in-One’ [91] and its updated version ‘Pse-in-One2.0’ [92] have been established that can be used to generate any desired feature vectors for protein/peptide and DNA/RNA sequences according to the need of users’ studies.

Here, we used a variety of sequence-derived features to generate various different modes of general PseAAC that have proven useful in our previous studies. These include:

1. Binary sequence profile feature (termed as BINARY), which refers to the encoding of amino acid sequences using the 21-bit (20 amino acid types plus a 21-th gap-filling residue ‘X’) binary encoding method, as previously described [37, 51]. For a local sliding window of 16 amino acids to encode a potential cleavage site, the dimensions of this feature type are 21 × 16 = 336.
2. Composition of *k*-spaced amino acid pair (CKSAAP) [54, 56–59], which was originally termed as collocated amino acid pair encoding [56, 57, 93]. This encoding reflects the short-range interactions of residues within the sequence surrounding potential cleavage sites [59]. Taking *k *= 0 as an example, there are 400 distinct types of 0-spaced amino acid pairs (i.e. AA, AC, AD, …, YY). Then, a feature vector can be defined as:
  (2) $${\left(\frac{N_{AA}}{N_{total}},\frac{N_{AC}}{N_{total}},\;\frac{N_{AD}}{N_{total}},\ldots ,\frac{N_{YY}}{N_{total}}\right)}_{400}.$$
  The value of each descriptor denotes the composition of the corresponding amino acid pair in the protein or peptide sequence. For example, if the amino acid pair AA appears *n* times in the sequence, the composition of the amino acid pair AA is equal to *n* divided by the total number of 0-spaced amino acid pairs ($N_{total}$) in the local sliding window. We defined *L* to be the length of the local sliding window of cleavage site. In this case, *L *=* *16, and the value of $N_{total}$ is *L*—(*k + *1). In this study, the CKSAAP encoding was performed over *k *=* *0, 1, 2, 3, 4 and 5. Thus, the dimension of the CKSAAP feature vector is 400 × 6 = 2400.
3. *K*-nearest neighbor (KNN) features, which describe the cluster information of local sequences for predicting potential sites [58, 60]. This feature type ranks the top *K* peptides by computing the similarity scores between the query peptide and all peptides in both the positive and negative sets [58]. The similarity score between two peptide sequences $P_{1}$ and $P_{2}$ is defined as:
  (3) $$Score=\overset{n}{\underset{i=1}{\sum }}S(P_{1,i},\;P_{2,i}),$$
  (4) $$S(a,b)=\{\begin{array}{c} BLOSUM62(a,b),\;if(BLOSUM62)>0\\ 0,\;if(BLOSUM62)\leq 0 \end{array},$$
  where *P* is the peptide with *n* amino acids, *i* is the amino acid position in the sequence and $BLOSUM62\left(a,b\right)$ is the corresponding element value for amino acids *a* and *b* in the BLOSUM62 matrix. Then, the ratio of positive samples in the top *K* peptides will be calculated. In this study, we set *K *= 1, 3, 5, 7 and 9% of the total numbers of positive and negative samples.
4. AAC, which is based on the calculation of the occurrence frequency of each of the 20 amino acid types in a local window. The frequencies of all 20 natural amino acids (i.e. ‘ACDEFGHIKLMNPQRSTVWY’) can be calculated as:
  (5) $$f(a)=\frac{N(a)}{L},\;a\in \{A,C,D,\ldots ,Y\},$$
  where N(a) is the number of occurrences of amino acid *a*, while *L* is the local window length. The dimension of the AAC feature vector is 20.

#### Physicochemical property features

These include: (i) charge/hydrophobicity ratio (termed as CHR) [59], which describes the charge and hydrophobicity ratio of the sequences surrounding cleavage sites; (ii) AAIndex features. AAindex [63] is a database of amino acid indices and amino acid mutation matrices. In the current version of the AAindex database (Version 9.2), 566 amino acid indices can be retrieved. Using the AAindex database, we extracted AAindex features that reflected the physicochemical properties of the sequences surrounding potential cleavage sites.

#### Evolutionary features

These include: (i) position-specific scoring matrix (PSSM) [61], which reflects the evolutionary information of the amino acids surrounding the cleavage sites; (ii) BLOSUM62. The BLOSUM62 matrix [62] is used to represent the sequence information surrounding a potential cleavage site, which reflects the similarity of two sequence fragments.

#### Structural features

In addition to the above features, we also incorporated structural information predicted from protein sequences, which include: (i) protein secondary structures predicted by SABLE [53]; (ii) solvent accessibility predicted by SABLE [53]; and (ii) natively disordered region predicted by DISOPRED2 [64].

Altogether, using a sliding window of 16 amino acids to encode and represent each potential cleavage site, we generated a 4562-dimensional feature vector based on the 11 types of features described above. Accordingly, each candidate cleavage site was represented by a feature vector *x* with 4562 feature components {*x*_1_, …, *x*_4562_}.

### Feature selection

To improve the feature representation ability and identify a subset of optimal features that contribute the most to the prediction of substrate cleavage sites, we used a two-step feature selection strategy, which combined mRMR [42] with forward feature selection (FFS) as described in our previous work [30, 44, 45, 94].

In this two-step feature selection strategy, the first step is to characterize the relative importance and contribution of each initial feature in the extracted feature set using the mRMR algorithm, which is able to rank all the initial features according to both their relevance to the response variables and the redundancy between the features themselves. Features that were assigned with higher ranking by mRMR were considered as having a better trade-off between their maximum relevance and minimum redundancy. After the first step, we selected the top 100 features as the optimal feature candidates (OFCs).

The second step is to apply the FFS method to sequentially select the most representative subset of optimal features from the 100 OFCs identified above. FFS adds a feature each time (usually starting with the feature that had the highest index assigned by mRMR, all the way to the feature that had the lowest index) and reconstruct the SVM model by performing the 5-fold cross-validation test. As a consequence, FFS resulted in a feature subset that led to the best predictive performance [measured by the area under the receiver operating characteristic (ROC) curve, AUC] of SVM models. The feature subset that resulted was then recognized as the optimal feature set. Finally, we obtained 38 protease-specific SVM models optimized by this two-feature selection strategy based on the benchmark training substrate data set for each protease.

### Machine learning methods

SVM is an efficient machine learning algorithm suitable for solving binary classification, multiple classification or regression problems. The version of SVM best suited to predicting numerical outcomes is support vector regression (SVR). In this application, we used SVR to construct the prediction model to estimate the cleavage probability of substrate cleavage sites for a given protease. Owing to its excellent generalization capabilities, SVR has recently been applied in a growing number of applications in bioinformatics and computational biology, including cleavage site prediction [15, 29, 30], residue accessible surface area [95], protein B-factor [96, 97], half sphere exposure [98], disulfide connectivity [99], residue depth [54], torsion angles [29] and protein expression-level prediction [100]. It demonstrates competitive performance compared with other machine learning approaches, especially when dealing with real-valued prediction tasks.

The SVR classifier is able to find a linear discriminative function of the form:

(6) $$f(x)=W^{T}\Phi (x)+w_{0},$$

where Φ is a basis function that maps the *D*-dimensional feature vector to a higher dimension. It is noteworthy that although $f\left(x\right)$ is a linear function of $\Phi \left(x\right)$, it can itself be a nonlinear function of *x***,** which reflects an attractive advantage of using kernel methods [101]. SVR assumes that the best discriminative function is the one that represents the largest separation or margin between the two classes of samples.

For implementation of the SVR algorithm, we used the LibSVM software package [102] with the regression mode. The model performance was fully evaluated by using 5-fold cross-validation and independent tests. The model parameters were optimized using the benchmark training data set, and the predictive performance of the SVR models for each protease was evaluated by performing 5-fold cross-validation using the benchmark data set and independent tests using the independent test data set. In particular, for each major sequence encoding scheme, we trained a corresponding SVR model. In addition, we have also concatenated all the initial features and generated an all feature-based model (referred to as ALL-Fea). We also performed feature selection experiments to identify a subset of optimal features for the cleavage site prediction of each protease, and accordingly trained a selected feature-based model (denoted mRMR-FS).

### Performance evaluation

To quantitatively evaluate the performance of a model, a set of four metrics is usually used in the literature. They include: (1) overall accuracy (Acc), (2) Mathew’s correlation coefficient (MCC), (3) sensitivity (Sn) and (4) specificity (Sp), as given below (see, e.g., [103]):

$$\{\begin{array}{ll} Sn=\frac{TP}{TP+FN} & \left(7\right)\\ Sp=\;\frac{TN}{TN+FP} & \left(8\right)\\ Acc=\;\frac{TP+TN}{TP+TN+FP+FN} & \left(9\right)\\ MCC=\;\frac{\left(TP\times TN\right)-\left(FP\times FN\right)}{\sqrt{\left(TP+FP\right)\;\left(TP+FN\right)\left(TN+FP\right)\left(TN+FN\right)}} & \left(10\right) \end{array}$$

where *TP*, *TN*, *FP* and *FN* denote the numbers of true positives, true negatives, false positives and false negatives, respectively.

However, the above four metrics copied from math books lack intuitiveness and are not easy-to-understand for biologists, particularly the MCC, which is an important metric used for describing the stability of a predictor. Further, based on the Chou’s symbols introduced in the study of protein signal peptides [104, 105], a set of four intuitive metrics was derived [106, 107], which are given below:

$$\{\begin{array}{lll} Sn=1-\frac{N_{-}^{+}}{N^{+}} & 0\;\leq Sn\leq 1 & \left(11\right)\\ Sp=1-\frac{N_{+}^{-}}{N^{-}} & 0\leq Sp\leq 1 & \left(12\right)\\ Acc=\;\Lambda =1-\frac{N_{-}^{+}+N_{+}^{-}}{N^{+}+N^{-}} & 0\leq Acc\leq 1 & \left(13\right)\\ MCC=\;\frac{1-\left(\frac{N_{-}^{+}}{N^{+}}+\frac{N_{+}^{-}}{N^{-}}\right)}{\sqrt{\left(1+\frac{N_{+}^{-}-N_{-}^{+}}{N^{+}}\right)\;\left(1+\frac{N_{-}^{+}-N_{+}^{-}}{N^{-}}\right)}} & -1\leq MCC\leq 1 & \left(14\right) \end{array}$$

where $N^{+}$ represents the total number of positive samples being investigated, while $N_{-}^{+}$ is the number of positive samples incorrectly predicted to be negatives; $N^{-}$ denotes the total number of negative samples being investigated, while $N_{+}^{-}$ denotes the number of the negative samples incorrectly predicted to be positives.

According to Equations (11)–(14), we can easily see the following: when $N_{-}^{+}=0$, Sn=1; while when $N_{-}^{+}=N^{+}$, we have Sn=0. Likewise, when $N_{+}^{-}=0$, Sp=1; while when $N_{+}^{-}=N^{-}$, Sp=0. When $N_{-}^{+}=N_{+}^{-}=0$, we have Acc=MCC=1; while when $N_{-}^{+}=N^{+}$ and $N_{+}^{-}=N^{-}$, Acc=1 and MCC=-1; when $N_{-}^{+}=N^{+}/2$ and $N_{+}^{-}=N^{-}/2$, MCC=0.

As we can see, based on the definition of Equations (11)–(14), the meanings of Sn, Sp, Acc and MCC have become much more intuitive and easier to understand, as concurred in a series of recent publications (see, e.g., [80, 84, 86, 88, 90, 106, 108–113]). It is instructive to point out that the performance metrics as defined in Equations (7)–(10) or Equations (11)–(14) are valid only for single label systems; whereas for multi-label systems (see, e.g., [114–117]), a set of more complicated metrics should be used as discussed in [118].

In addition, the value of AUC (under ROC curve) [119] was also used to quantitatively measure the quality of the predictors in this package via the 5-fold cross-validations and independent data set tests.

## Results and discussion

### Performance evaluation based on different sequence encoding schemes

**Figure 2:**
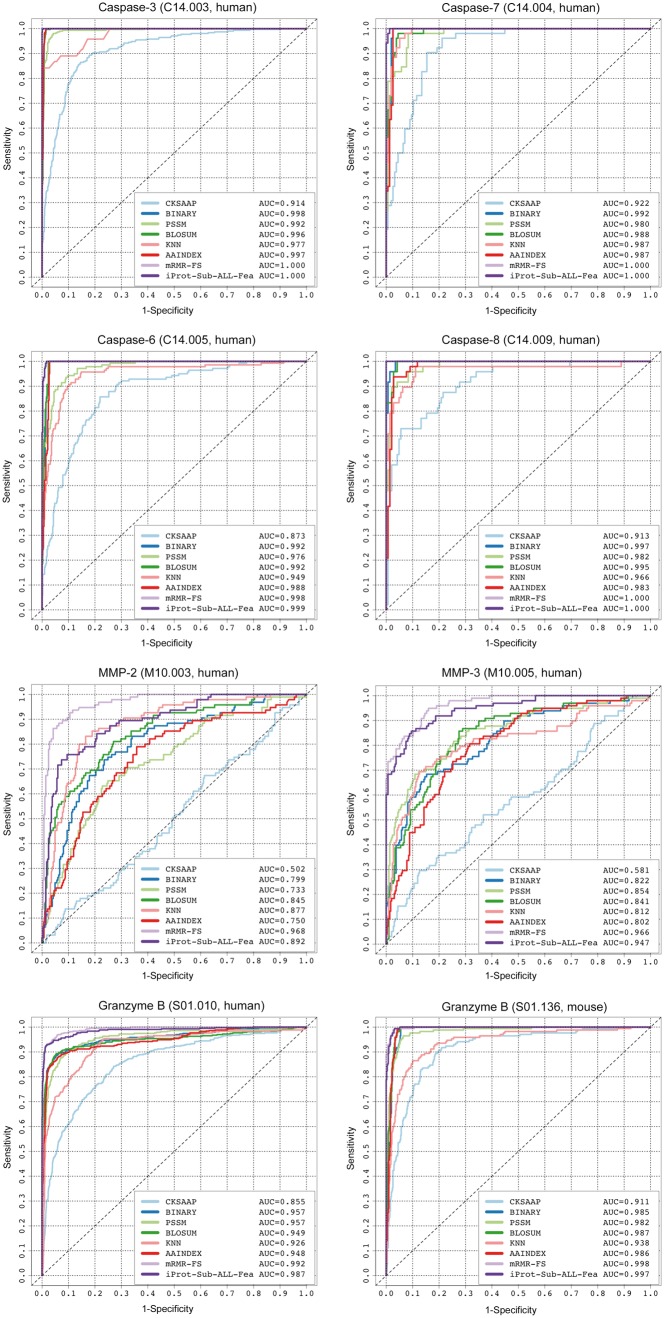
ROC curves of iProt-Sub models trained using different encoding schemes and their combinations for cleavage site prediction of eight proteases on the 5-fold cross-validation test.

In this section, we investigate the predictive performance of SVR models using different sequence encoding schemes and their combinations for cleavage site prediction of multiple proteases, by performing 5-fold cross-validation. The compared sequence encoding schemes include ‘BINARY’, ‘PSSM’, ‘BLOSUM’, ‘KNN’, ‘CKSAAP’ and ‘AAIndex’. In addition, we also compared the performance of SVR models that were trained using all the initial features (referred to as ‘ALL-Fea’) and optimal selected features (termed ‘mRMR-FS’) after the two-step mRMR-FS feature selection. The ROC curves of these SVR models for cleavage site prediction of eight proteases [caspase-3, -6, -7, -8, MMP-2, -3, granzyme-B (human) and granzyme-B (mouse)] on the 5-fold cross-validation are shown in Figure 2.

Several important observations can be made. First, we can see that the ‘ALL-Fea’ model and ‘mRMR-FS’ model generally outperformed the other six models trained based on individual encoding schemes, with the AUC values ranging between 0.89 and 1.0. Second, the ‘mRMR-FS’ model achieved the overall best performance, after the two-step feature selection, compared with the other models for the MMP cleavage site prediction. For example, the ‘mRMR-FS’ model achieved an AUC of 0.968 for MMP-2 cleavage site prediction, while the second best ‘ALL-Fea’ model achieved an AUC of 0.892. Third, the accuracy of protease-specific cleavage site prediction varies substantially between different proteases and different protease families. The difficult cases include cleavage site prediction of the MMP family and other proteases (e.g. thrombin) whose activities are also regulated by confounding factors such as the presence of exosites (sites that are located outside the active sites) [120–122]. Compared with the caspases and granzyme B, the performance of cleavage site prediction with the MMP family achieved by a model using the same encoding scheme is much worse in terms of the AUC score. For example, the CKSAAP model only achieved an AUC of 0.502 and 0.581 for the cleavage site predictions of MMP-2 and MMP-3, respectively, compared with that of 0.914 and 0.922 for the cleavage site prediction of caspase-3 and caspase-7, respectively (Figure 2). Future studies should investigate incorporation of other relevant features that might prove useful for improving the predictive performance of cleavage sites for proteases with a requirement for allosteric regulation to cleave their target substrates.

### Amino acid distributions in substrate cleavage site

**Figure 3:**
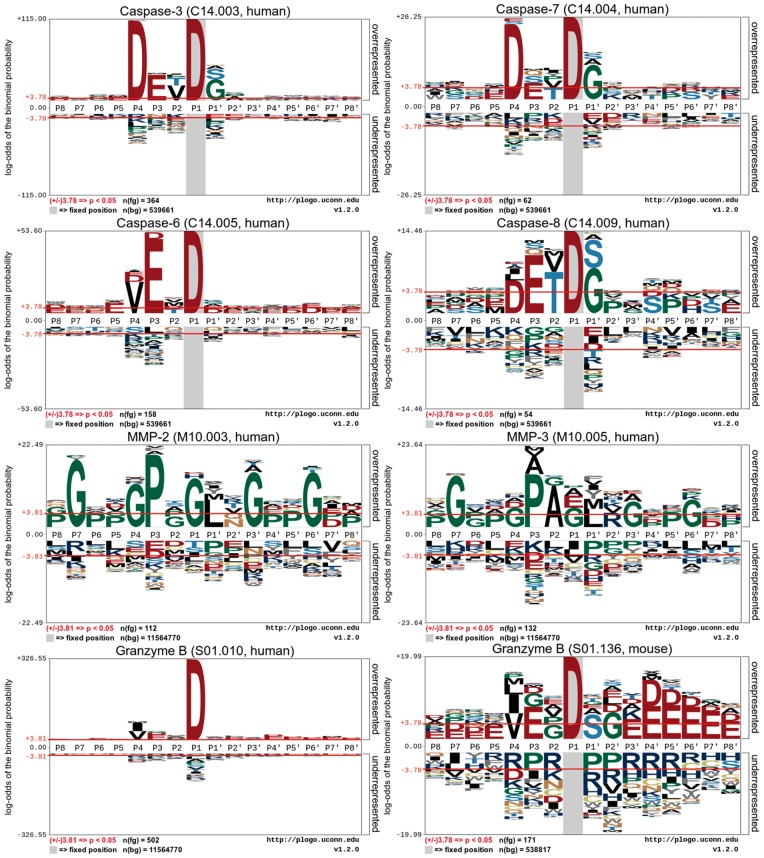
Sequence logo representations of experimentally verified cleavage sites (P8–P8′) of eight proteases caspase-3, -6, -7, -8, MMP-2, 3, granzyme-B (human) and granzyme-B (mouse). Sequence logos were generated using pLogo and scaled to the height of the largest column within the sequence visualization. The red horizontal lines on the pLogo graph denote the threshold of *P* = 0.05.

To better understand informative features surrounding a cleavage site that may define protease-specific substrate cleavage, we examined the flanking sequences of protease-specific substrate cleavages with the pLogo program [123], a probabilistic approach to identifying the presence and visualization of sequence motifs. The generated sequence logo diagrams for caspase-3, -7, -6, -8, MMP-2, MMP-3, granzyme B (human) and granzyme B (mouse) are shown in Figure 3. To perform the sequence logo analysis, we examined the P8–P8′ sites according to the Schechter–Berger nomenclature [124]).

Indeed, the sequence logos in Figure 3 show that there exist conserved sequence motifs or distinctive sequence patterns surrounding protease-specific substrate cleavage sites that may potentially be used to differentiate between different proteases. Notably, it can be seen that a predominant characteristic of substrate cleavage sites of caspases (caspase-3, -6, -7 and -8) is the requirement of Asp residue at the P1 position [125]. For certain caspases (e.g. caspase-3 and -7), there is also a lesser selectivity for Asp residue at the P4 position, thereby constituting the canonical DXXD motif [125]. A commonality of the cleavage selectivity of granzyme B, compared with that of caspases, is that they primarily recognize and cleave after the Asp at the P1 position as well. On a closer look, we can see that there exist subtle differences in the substrate cleavage selectivity between granzyme B (human) and granzyme B (mouse) [126]. Apparently, granzyme B (mouse) has a more complicated preference favoring a number of residue types across different positions surrounding the cleavage sites, including Val, Pro and Gly at the P1 position; Ser at the P1′ position; and Gly at the P2′ position, respectively, while granzyme B (human) has much less selectivity at these positions.

However, different from caspases, matrix metallopeptidases (e.g. MMP-2 and MMP-3) have distinctive substrate specificities (Figure 3). Specifically, Gly was significantly overrepresented at the P7, P4, P2, P1, P3′ and P6′ positions surrounding the cleavage sites (*P* < 0.05, Figure 3). Owing to the intriguing selectivity of multiple residue types across different positions, it is much more difficult to clearly define distinctive sequence motifs for the MMPs. These results highlight the importance and need to improve the substrate cleavage site prediction by developing more accurate machine learning-based predictors, especially for proteases for which canonical sequence motif-based methods fail to perform well.

### Feature contribution analysis

**Figure 4:**
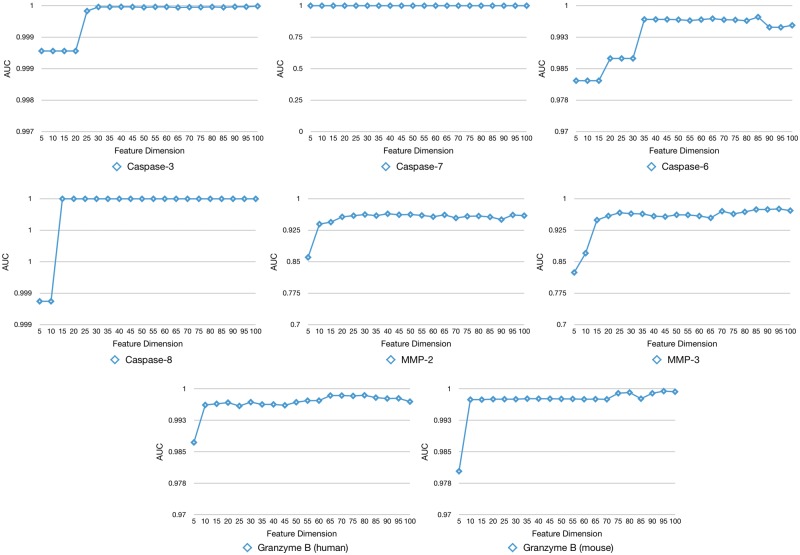
The feature selection curve in stepwise feature selection describes the performance change (in terms of AUC) as a function of the number of gradually increased OFCs.

We used a two-step feature selection strategy by combining the mRMR algorithm [36] with FFS to characterize a subset of optimal features that contributed the most to the the prediction of substrate cleavage sites of each protease. Figure 4 shows the performance change (in terms of the AUC value) of the trained SVR models by gradually adding the selected features in a step-wise manner. As can be seen, all the feature selection curves started with quickly increasing the AUC value and then settled into the plateau after reaching their maximum at the peak, while in some cases, adding more features will lead to a drop in the AUC value (Figure 4).

Because 11 different types of features were originally extracted and used for training the models, it is of particular interest to characterize their relative importance and contribution to cleavage site prediction performance. In the ‘ALL-Fea’ sequence encoding scheme that encoded all the initial features, 11 different types of features were included. After the two-step feature selection based on mRMR and FFS, seven types of features remained in the final optimal feature subset of cleavage site prediction for eight proteases. To evaluate the contribution of these seven different feature types to the classification performance for individual proteases, the performance difference can be measured using the AUC value when a particular feature type is removed from the classifier. This measure thus represents the additional value of such feature type in cleavage site prediction, accounting for both interaction and compensatory effects between features [127]. Here, we define this measure as the ‘contribution percentage’ for a feature type by calculating the percentage of AUC decrease relative to other feature types after removing the feature from the classifier.

**Figure 5:**
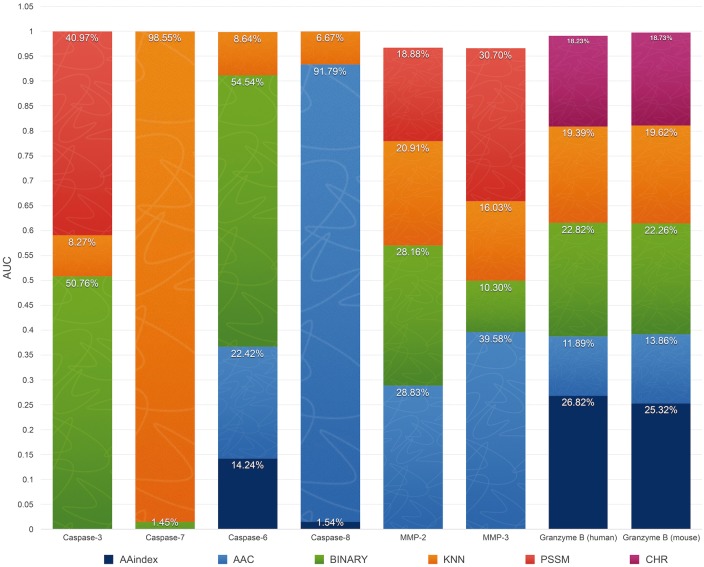
Importance of different feature types to the improvement of cleavage site prediction performance for eight proteases. The height of each bar for a feature type represents the proportional ‘contribution percentage’ that represents the AUC value of the feature selection model for one protease, and the uniformed AUC drop rate for each type of feature is represented with different colors. The AUC drop rate was obtained by comparing with the model after removing this feature from the input.

From Figure 5, we can see that the three most important types of features are KNN features with a contribution percentage ranging from 6.67% (for caspase-8) to 98.55% (for caspase-7), AAC features with a contribution percentage ranging from 1.45% (for caspase-7) to 54.54% (for caspase-6) and BINARY features with a contribution percentage ranging from 1.45% (for caspase-7) to 54.54% (for caspase-6). Among these three feature types, KNN features appear to be essential and thus most important for the predictive performance, as it was included in the feature sets of all proteases under investigation. In addition, the feature importance also varies depending on the protease, for example KNN features made an exclusive contribution to caspase-7 cleavage site prediction, but only made a moderate contribution to caspase-3 cleavage site prediction, secondary to BINARY and PSSM features. This is also the case for AAC features, which made a predominant contribution to caspase-8 cleavage site prediction (with a contribution percentage of 91.79%), but a marginal contribution to granzyme B (human and mouse) (with contribution percentages of 11.89 and 13.86%, respectively) cleavage site predictions, and completely dropped out of the final optimal feature subsets in the case of caspase-3 and -7 (Figure 5).

### Performance comparison between iProt-Sub and other general prediction tools

**Figure 6:**
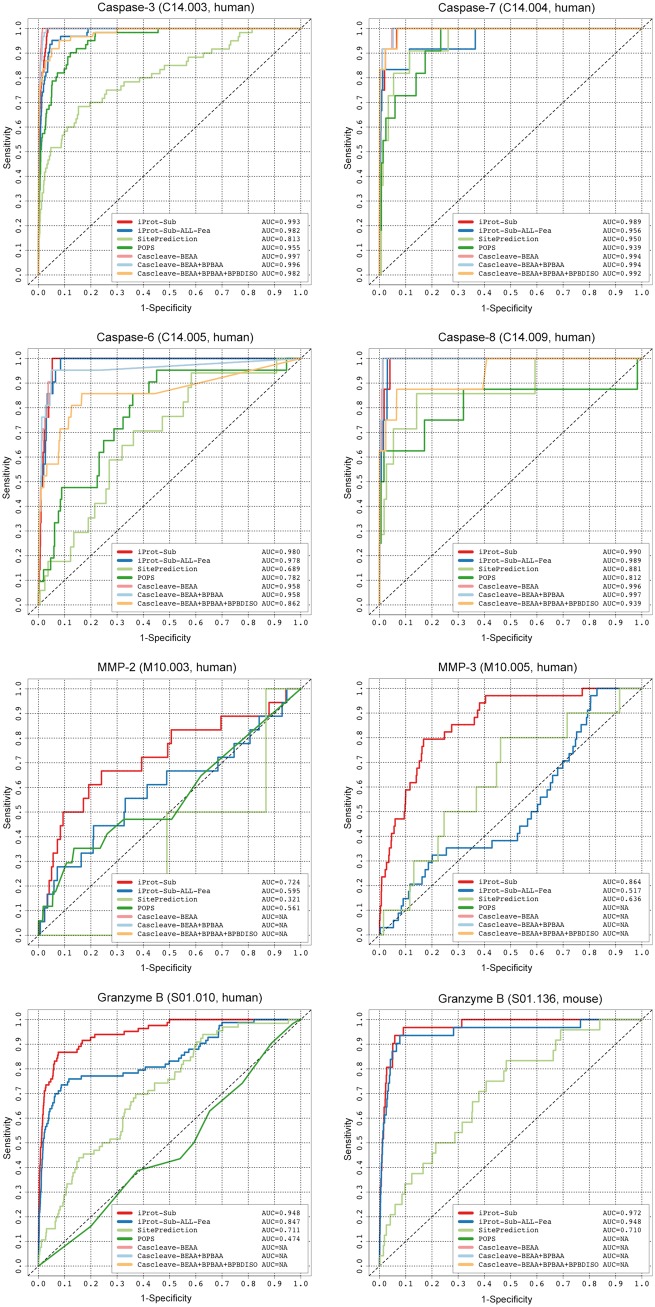
Performance comparison between iProt-Sub and other existing methods for cleavage site prediction for different proteases based on the independent test data sets, evaluated using ROC curves.

In this section, we performed an independent test and compared the performance of iProt-Sub with three state-of-the-art general prediction tools that can be used to predict the substrate cleavage sites for multiple proteases: PoPS [33], SitePrediction [34] and Cascleave [15]. As a number of other tools were developed for specific proteases *per se*, they were not included in this comparative analysis. In addition, as the compared tools use different training data and algorithms to develop their respective prediction rules/models, the predictive capability of these tools differs from each other. Thus, to avoid any potential bias, for a protease, we only compared with tools that could provide valid prediction results after submitting the sequences of the independent test data set to each of the online Web servers. As a result, the ROC curves and calculated AUC values of cleavage site prediction for caspase-3, -6, -7, -8, MMP-2, MMP-3, granzyme B (human) and granzyme B (mouse) are shown in Figure 6.

It is of particular interest to compare the performance of iProt-Sub with Cascleave, which also uses the SVR algorithm and sequence-derived features (such as BINARY, predicted secondary structure and native disorder information) to train the prediction models. The online Web server of Cascleave provides three model options: Cascleave-BEAA, Cascleave-BEAA + BPBAA and Cascleave-BEAA + BPBAA + BPBDISO, which were trained using three different sequence encoding schemes [15]. PoPS is one of the most popular bioinformatics tools for modeling and predicting substrate specificity. It creates a simple matrix-based specificity model with different weights for amino acid residues at different positions, built from experimental data or expert knowledge and available to the user [33]. The specificity model can be used to score, predict and rank likely cleavage sites within a given substrate sequence for the designated protease of interest. SitePrediction is also a general prediction tool for predicting cleavage sites in candidate substrates. To make an accurate prediction, SitePrediction also considers other additional features that describe the environment of potential cleavage sites (including solvent accessibility, secondary structure and sequence similarity to the known cleavage sites) in conjunction with the amino acid frequency scores [34]. Both PoPS and SitePrediction are regarded as statistical scoring function-based tools, while Cascleave and iProt-Sub are considered as machine learning-based tools.

As can be seen from Figure 6, iProt-Sub achieved the overall best predictive performance compared with the other three tools PoPS, SitePrediction and Cascleave (with three models) for six proteases [including caspase-3, 6, MMP-2, MMP-3, granzyme B (human) and granzyme B (mouse)], with the only exception of caspase-7 and -8, for which iProt-Sub performed the second best, with an AUC of 0.989 and 0.990, respectively, in contrast to the best-performing tool Cascleave, which achieved an AUC of 0.994 and 0.997, respectively. For those proteases that iProt-Sub achieved the best performance, its performance gains over the other compared tools are apparent, which is particularly the case for MMP-2, MMP-3, granzyme B (human) and granzyme B (mouse) (Figure 6).

In addition, we note that although the strategy incorporating additional features generally improved the cleavage site prediction performance for some proteases [e.g. caspase-7, MMP-3, granzyme B (human),] in combination with feature selection, it decreased the performance for other proteases [e.g. caspase-3, -6, -8 and granzyme B (mouse)]. This can be observed by comparing the ROC curves and AUC values between the iProt-Sub models and iProt-Sub-ALL-Fea models in Figure 6. The underlying reason for this outcome is not entirely clear but might be associated with the size of the cleavage site data sets and the presence of other confounding factors that influence the cleavage outcome.

Overall, the results of the independent test indicate that by integrating heterogeneous informative features selected by an effective two-step feature selection strategy coupled with the SVR algorithm, iProt-Sub is able to provide a competitive predictive performance of substrate cleavage site prediction when compared with three existing prediction tools.

### The implementation of iProt-Sub Web server

To facilitate high-throughput prediction and analyses of novel protease-specific substrates and cleavage sites, we have implemented an online Web server of iProt-Sub for the wider research community to use. The Web server was designed with a user-friendly interface and modern data visualization functionality and is freely available at http://iProt-Sub.erc.monash.edu/. It was implemented using Java Server Pages running Tomcat7 and configured in the Linux environment on a 16-core server machine with 50 GB memory and a 4 TB hard disk. To submit a prediction job, the server requires protein amino acid sequences (the submission of up to 50 sequences is permitted simultaneously) in FASTA format as the input. Users are also required to provide their e-mail addresses to receive a notification e-mail that contains a link to the prediction output Web page after the submitted job is completed. For a protein sequence with 500 amino acid residues, the prediction task will generally take approximately 3 min to calculate the features and return the final prediction results. A step-by-step guideline of how to use the iProt-Sub Web server can be found at http://iProt-Sub.erc.monash.edu.au/help.html.

**Figure 7:**
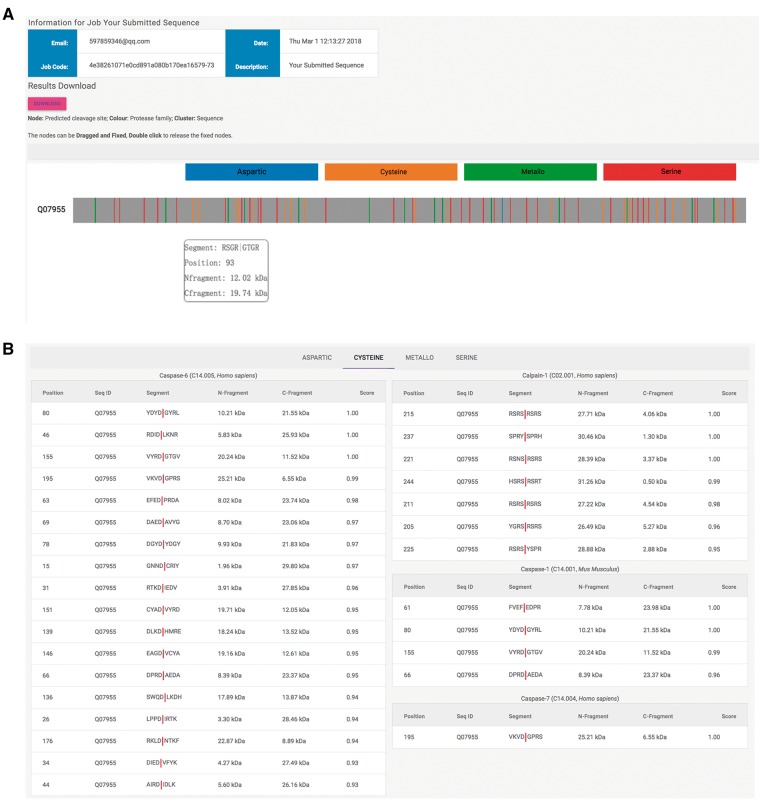
Example output of the iProt-Sub Web server.

Figure 7 provides an example output of the Web server. As can be seen, there are two main sections of the prediction output involving graphical visualization output (Figure 7A) and ranking output (Figure 7B) of the predicted cleavage sites in a protease family-specific manner. In terms of the graphical output, all the predicted cleavage sites are indicated by vertical lines with different colors (different colors indicate a different protease family, such as aspartic, cysteine, metallo, and serine). When hovering the mouse cursor over each differentially colored vertical line, a window pops up displaying detailed information associated with the predicted cleavage site/outcome, including the P4–P4′ sequence segment, cleavage site P1 position and the estimated sizes of N- and C-fragment cleavage products (Figure 7A). This graphical visualization function can greatly facilitate the quick identification of the predicted cleavage site(s) of interest by scanning from the N-terminus to C-terminus and visually comparing the cleavage profiling within the same substrate sequence across different proteases. The ranking output provides a tab-style view of the predicted cleavage sites according to the protease family (Figure 7B). Each tab contains the residue position of the predicted cleavage P1 site, the sequence ID, P4–P4′ sequence segment (with the predicted cleavage site indicated by ‘|’), the estimated N- and C-fragment sizes and the cleavage probability score.

Features used by the Web server for predicting cleavage sites include 11 previously mentioned feature encoding schemes, such as BINARY, AAC, PSSM, AAIndex, BLOSUM, CHR, CKSAAP, SS, SA, DISO and KNN. Based on the functionalities mentioned above, iProt-Sub offers important advantages over existing prediction servers in its ability to identify potential substrates and achieves a greater coverage and accuracy than previous predictors. To our knowledge, iProt-Sub is the most comprehensive server that is capable of predicting substrate cleavage sites of multiple proteases within a single substrate sequence using machine learning techniques. It is anticipated to be a valuable tool for cost-effective *in silico* identification of novel protease-specific substrates and cleavage sites.

### Proteome-wide prediction and Gene Ontology enrichment analysis of protease-specific substrates at the proteome level

We applied the developed iProt-Sub tool to scan the human proteome (149 730 proteins) with a high stringency at the 100% specificity level in an effort to provide an overview of the substrate repertoire of several important proteases and gain insights into the significantly enriched Gene Ontology (GO) [128] terms and biological pathways of these ‘computational’ substrates at the entire human proteome level. Seven protease-specific models [caspase-3, -6, -7, -8, MMP-2, -3 and granzyme B (human)] were used to conduct the human proteome-wide scan, and >20 200 reviewed human protein sequences downloaded from the UniProt database were involved in this analysis. Note that to generate highly accurate mapping, we applied the prediction models that were trained using the final optimal features based on the complete training data sets to perform the proteome-wide substrate scanning. The statistics for the predicted substrate proteins and cleavage sites are shown in Table 3. A complete list of the predicted substrates for each protease and their corresponding substrate cleavage sites is available from the iProt-Sub website.

**Table 3.** Statistical summary of the predicted substrates and cleavage sites with the 100% specificity at the proteome scale

MEROPS ID	Protease name	Number of predicted substrates	Number of predicted cleavage sites
C14.003	Caspase-3	10 645	26 929
C14.004	Caspase-7	12 288	34 355
C14.005	Caspase-6	5936	10 156
C14.009	Caspase-8	18 532	152 609
M10.003	MMP-2	9805	22 985
M01.005	MMP-3	402	425
S01.010	Granzyme B (human-type)	13 995	47 092

**Figure 8:**
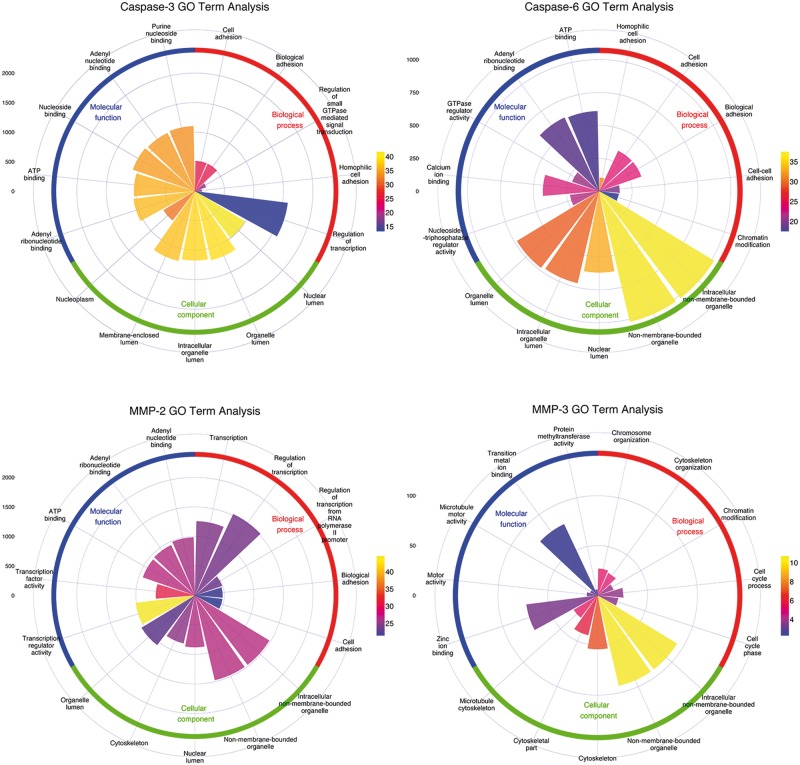
Functional enrichment analysis of the predicted substrates for caspase-3, -6, MMP-2 and -3 at the proteome scale, according to the BP, CC and MF classifications of GO terms. The statistical enrichment analyses of GO terms for predicted substrates were performed with the hypergeometric distribution.

**Figure 9:**
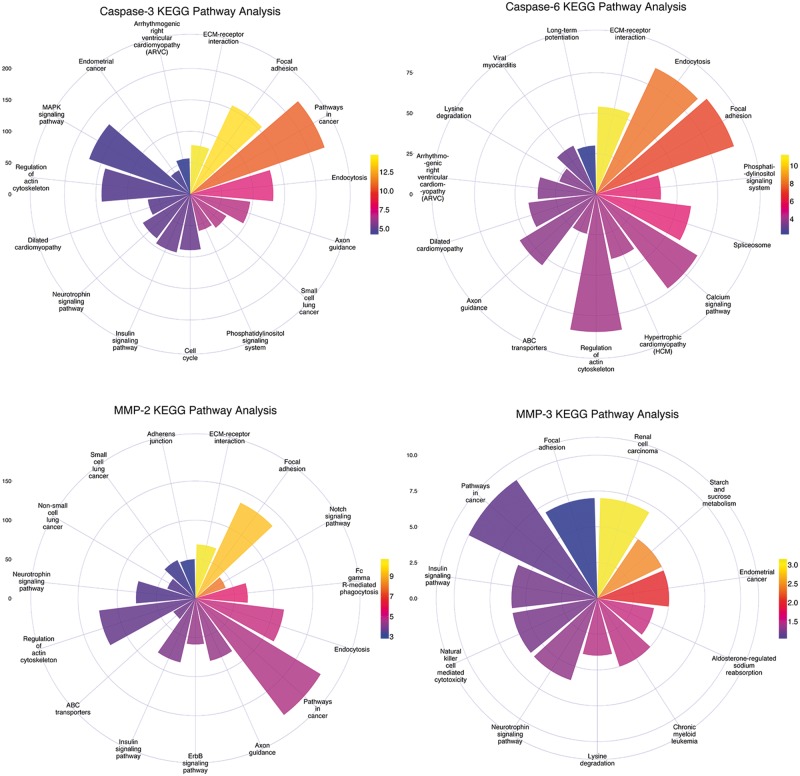
The KEGG pathway enrichment analysis of the predicted substrates for caspase-3, -6, MMP-2 and -3 at the human proteome scale. The statistical enrichment analyses of GO terms for predicted substrates were performed with the hypergeometric distribution.

Based on the proteome-wide scanning results, we further conducted an enrichment analysis using the DAVID online server [129], including GO analysis and KEGG pathway analysis. The top five significantly overrepresented biological process (BP), cellular component (CC), and molecular function (MF) terms, and KEGG pathways of the predicted substrate proteins for caspase-3, caspase-6, MMP-2 and MMP-3 at the proteome scale are highlighted in Figures 8 and 9, respectively. The sectorial area for a GO term represents the number of proteins with this term, while the different colors of the sectorial area indicate the statistical significance of the enrichment for the corresponding GO term. In general, substrate proteins targeted by different protease families tend to be associated with different GO terms, but substrate proteins within the same family share similar GO terms. For example, both caspase-3 and caspase-6 substrates were found to be enriched with the GO BP terms ‘Cell adhesion’ and ‘Biological adhesion’ and with the GO MF terms ‘ATP binding’ and ‘adenyl ribonucleotide binding’. Similar tendencies can also be observed between the MMP-2 and MMP-3 substrates. With regard to the CC terms, many of the predicted substrates were found to be located in different components, including ‘Nuclear lumen’, ‘Intracellular organelle lumen’, ‘Organelle lumen’ and ‘Cytoskeleton’, where many apoptotic morphological changes and cellular signaling activities often occur [130].

In terms of pathway enrichment analysis, we observed that caspase and MMP substrates were highly enriched in several KEGG pathways that involve ‘Focal adhesion’, ‘ECM-receptor interaction’, different types of signaling pathways and ‘Pathways in cancer’ (Figure 9), highlighting the functional roles of these protease–substrate interactions in cancer-related biological processes. In addition, there also exist specific signaling pathways and cancer-related pathways that were specifically enriched for caspase-3 substrates (small cell lung cancer), caspase-6 substrates (hypertrophic cardiomyopathy), MMP-3 substrates (both small and non-small cell lung cancer) and MMP-6 substrates (endometrial cancer and chronic myeloid leukemia). These results highlight the functional roles of these protease–substrate interactions in cancer-related biological processes [1, 4, 5].

### Case study

To illustrate the predictive power of iProt-Sub, we performed a case study where the targeted cleavage of the protein calpastatin by caspase-3 [131] and MMP-2 [132] was examined in detail. Calpastatin (UniProt ID: P20810) is an endogenous calpain (calcium-dependent cysteine protease) inhibitor, which is encoded by the CAST gene in humans. It consists of an N-terminal domain and four repetitive calpain-inhibitory domains (Inhibitory Domains 1–4). It has been suggested that calpastatin is involved in the control of proteolysis of amyloid precursor protein and also in muscle protein degradation in living tissue [133].

**Figure 10:**
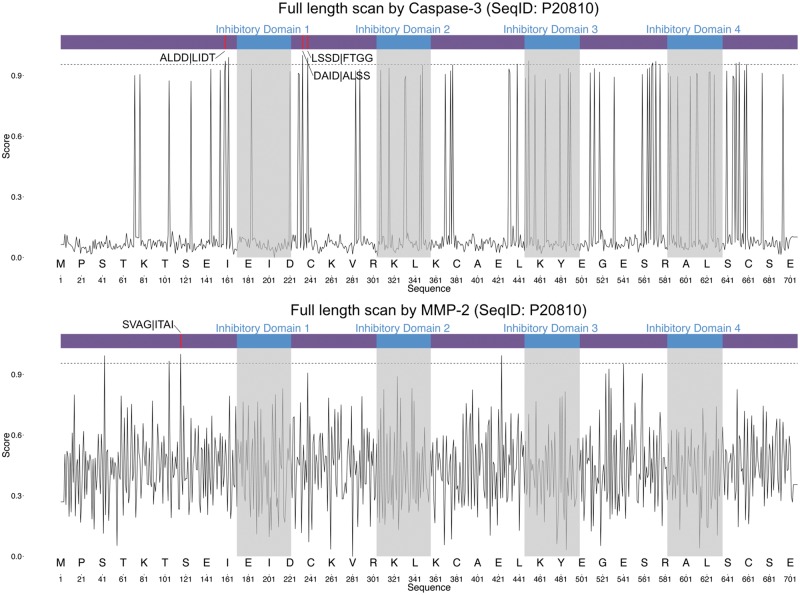
Full-length sequence scanning of calpastatin by iProt-Sub for caspase-3 (above) and MMP-2 cleavage sites (below). The horizontal axis denotes the amino acid residue position, while the vertical axis denotes the cleavage probability score generated by iProt-Sub. A higher threshold value of 0.95 is applied to identify the high-confidence cleavage site predictions, denoted by the dashed line. P4–P4′ sites surrounding the predicted cleavage P1 position are given.

Applying iProt-Sub to perform the substrate sequence scanning to calpastatin, we correctly identified all the three experimentally verified cleavage sites for caspase-3 [131]: ALDD|LIDT, DAID|ALSS and LSSD|FTGG (Figure 10). In terms of MMP-2 cleavage sites, we also identified an additional cleavage site for MMP-2: SVAG|ITAI [132]. Note that all these experimentally verified cleavage sites were on the top-ranking list of hits according to the predicted probability score generated by iProt-Sub, and all were above the threshold of 0.95. Moreover, iProt-Sub-based substrate sequence scanning also led to the prediction of several other high-confidence novel potential cleavage sites for both caspase-3 and MMP-2 (Figure 10). These predicted cleavage sites may represent novel sites targeted for cleavage under different conditions, and require follow-up experimental validation and hypothesis-driven studies. All the results above highlight the usefulness and value of using iProt-Sub as an *in silico* tool for identifying novel putative cleavage targets and unraveling the protease–substrate interaction relationship.

### Limitations and future work

Despite the strong performance of our developed computational approach for predicting the substrate cleavage sites of multiple proteases, it has the following limitations:

The first limitation is that iProt-Sub is a machine learning-based approach and as such, its predictive power derives from the machine learning models that are trained based on different forms of sequence encoding schemes. The performance of machine learning-based models primarily depends on the effective representation of such feature encoding schemes. Accordingly, it remains a significant challenge in the future to identify further useful encoding schemes. There is much promise in this aspect from the availability of some recently developed powerful toolkits and Web servers for extracting a wide range of features, including Pse-Analysis [87], Bio-Seq Analysis [85], Pse-in-One [91], repDNA [81] and iFeature [134]. These toolkits could enable us to consider a much greater combination of different types of feature encoding schemes and explore the possibility of evolving iProt-Sub to a more robust framework while preserving or enhancing its model accuracy.

The second limitation is that the cleavage site prediction performance of iProt-Sub varies greatly between proteases. Its accuracy is lowest for matrix metalloproteases, including MMP-2 and MMP-3. The current study and several previous studies [29, 52, 135] confirmed the prediction of cleavage sites for these proteases to be an especially challenging problem and highlighted the need to develop specialized methods for improved MMP cleavage site prediction.

The third limitation is that most of the substrate cleavage sites used for training the prediction models of iProt-Sub were identified by high-throughput mass spectrometry methods, which might introduce a potential bias in terms of representing the global proteolytic events [9] and hence might impact on the predictive performance of the trained models [136]. Therefore, when sufficient heterogeneous cleavage site data sets identified by other different experimental approaches are available in the future, it will be important to characterize their potential influence on the predictive performance of cleavage sites.

The fourth limitation is that iProt-Sub only used the SVR algorithm to build the probabilistic cleavage site prediction models. In the future work, we plan to consider using other advanced machine learning techniques such as deep learning (DL), which can model high-level abstraction in the data [137], using significantly enlarged benchmark data sets (the next MEROPS release) to evaluate the performance of the DL models against other popular machine learning classifiers.

## Conclusions

We have developed the iProt-Sub tool and constructed protease-specific prediction models for 38 proteases. iProt-Sub substantially upgrades the PROSPER Web server, includes a user-friendly interface and provides users with easy-to-understand data visualization techniques to better serve the wider research community. We have conducted a comprehensive set of experiments to benchmark the performance of different sequence encoding schemes and compare the models with other previously proposed state-of-the-art methods. Our experimental evaluations indicate that the proposed iProt-Sub method outperforms those previously developed methods. iProt-Sub’s improved performance can be attributed to several important aspects. First, we have compiled a comprehensive experimentally verified cleavage site data set in this work. Second, protease-specific models have been constructed, optimized and validated to achieve a better performance than other available tools using the powerful SVR algorithm coupled with a two-step feature selection procedure. Third, iProt-Sub allows high-throughput prediction of potential substrate cleavage sites for follow-up experimental validation and hypothesis-driven functional studies. A unique feature of iProt-Sub is that, unlike previously developed tools that require users to designate the protease of interest to make the prediction, iProt-Sub for a given substrate sequence will identify which, if any, of its 38 proteases will cleave that substrate. This unique feature makes iProt-Sub an attractive tool for proteomic research, especially in cases where there is insufficient knowledge about the protease(s) responsible for such cleavage to occur. We expect that iProt-Sub will be used as a valuable and powerful tool by the protease community and can deliver vital functional clues regarding the protease–substrate interactivity relationship in a cost-effective manner.

###

**Key Points**

- In this work, we present iProt-Sub, a powerful bioinformatics tool for the accurate prediction of protease-specific substrates and their cleavage sites.
- It provides optimized cleavage site prediction models with better predictive performance and coverage for four major protease families and 38 proteases.
- iProt-Sub integrates heterogeneous sequence and structural features derived from multiple levels in combination with an effective two-step feature selection procedure.
- Benchmarking experiments using cross-validation and independent tests showed that iProt-Sub was able to achieve a better performance than several existing generic tools. It is publicly accessible at http://iProt-Sub.erc.monash.edu.au/.
- Application of iProt-Sub to scan the entire human proteome provides an insightful overview of the substrate repertoire of several important proteases and significantly enriched GO terms and biological pathways of the ‘computational’ substrates at the proteome level.

## Funding

This work was financially supported by grants from the Australian Research Council (ARC) (grant numbers LP110200333 and DP120104460), the National Health and Medical Research Council of Australia (NHMRC) (grant number 4909809), the National Institute of Allergy and Infectious Diseases of the National Institutes of Health (grant number R01 AI111965) and a Major Inter-Disciplinary Research (IDR) project awarded by Monash University.

## Notes

**Jiangning Song** received his BEng and DEng degrees from Jiangnan University, China. He is affiliated with the Monash Centre for Data Science, Faculty of Information Technology, Monash Biomedicine Discovery Institute and ARC Centre for Excellence in Advanced Molecular Imaging, Monash University, Melbourne, Australia. His research interests include bioinformatics, computational biology, machine learning, data mining and pattern recognition.

## Notes

**Yanan Wang** received his MEng degree from Shanghai Jiao Tong University, China. His research interests are bioinformatics, machine learning, data mining and pattern recognition.

## Notes

**Fuyi Li** received his BEng and MEng degrees in Software Engineering from Northwest A&F University, Yangling, China. He is currently a PhD student at the Biomedicine Discovery Institute, Monash University, Melbourne, Australia. His research interests are computational biology, bioinformatics, machine learning and data mining.

## Notes

**Tatsuya Akutsu** received his DEng degree in Information Engineering in 1989 from University of Tokyo, Japan. Since 2001, he has been a professor in the Bioinformatics Center, Institute for Chemical Research, Kyoto University, Japan. His research interests include bioinformatics and discrete algorithms.

## Notes

**Neil D. Rawlings** received his bachelor degree in Biological Sciences from Aston University, Birmingham, UK and his PhD from the Open University, Milton Keynes, UK. Since 1996, he and Alan J. Barrett have been the originators, developers and curators of the MEROPS database of proteolytic enzymes, their inhibitors and substrates, which provides the globally accepted classification of proteolytic enzymes and their inhibitors. He is one of the editors of the *Handbook of Proteolytic Enzymes* (Elsevier, 2013), and he is currently a curator for the InterPro database at the European Bioinformatics Institute, Cambridge, UK.

## Notes

**Geoffrey I. Webb** received his PhD degree in 1987 from La Trobe University, Australia. He is Director of the Monash Centre for Data Science and Professor in Faculty of Information Technology at Monash University, Australia. He is a leading data scientist and the only Australian to have been Program Committee Chair of the two leading Data Mining conferences, ACM SIGKDD and IEEE ICDM. He received the 2016 Australian Computer Society's ICT Researcher of the Year Award, the 2016 Australasian Artificial Intelligence Distinguished Research Contributions Award, a 2014 Australian Research Council Discovery Outstanding Researcher Award and the 2013 IEEE ICDM Service Award and was elevated to IEEE Fellow in 2015. His research interests include machine learning, data mining, computational biology and user modeling.

## Notes

**Kuo-Chen Chou** received his DSc degree in 1984 from Kyoto University, Japan. He is the founder and chief scientist of Gordon Life Science Institute. He is also a Distinguished High Impact Professor and Advisory Professor of several Universities. His research interests are in computational biology and biomedicine, protein structure prediction, low-frequency internal motion of protein/DNA molecules and its biological functions, diffusion-controlled reactions of enzymes, as well as graphic rules in enzyme kinetics and other biological systems.
